# Supplementary material for: Single drop cytometry onboard the International Space Station
Source: Nat Commun. 2024 Mar 25;15:2634. doi: 10.1038/s41467-024-46483-6 (PMC10963801; doi:10.1038/s41467-024-46483-6)
Supplement: Supplementary file 1 — Supporting Information [file 41467_2024_46483_MOESM1_ESM.pdf]

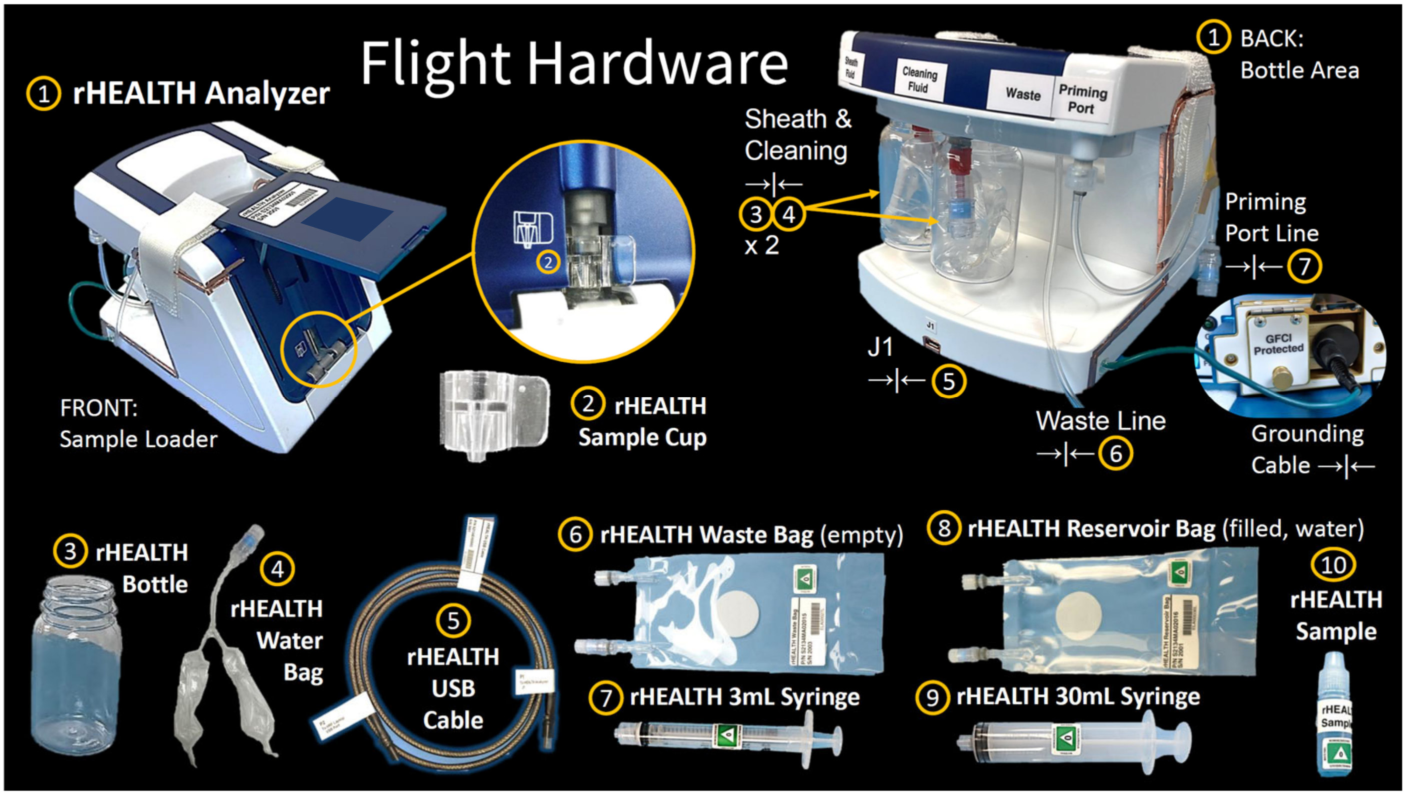


**Supplementary Fig. 1.** ***Detailed view of the flight hardware.*** The flight kit for the rHEALTH ONE Analyzer (1, logo digitally covered in dark blue on the door) included the following: rHEALTH sample cup (or sample consumable) (2), plastic bottles for sheath and cleaning (3), flexible water bag for inside of sheath and cleaning bottles (4), micro-USB cable and connection (5), waste bag and connection (6), burp syringe and connection (7), master rHEALTH reservoir bag (8), 30 mL syringe (9), and samples (10).

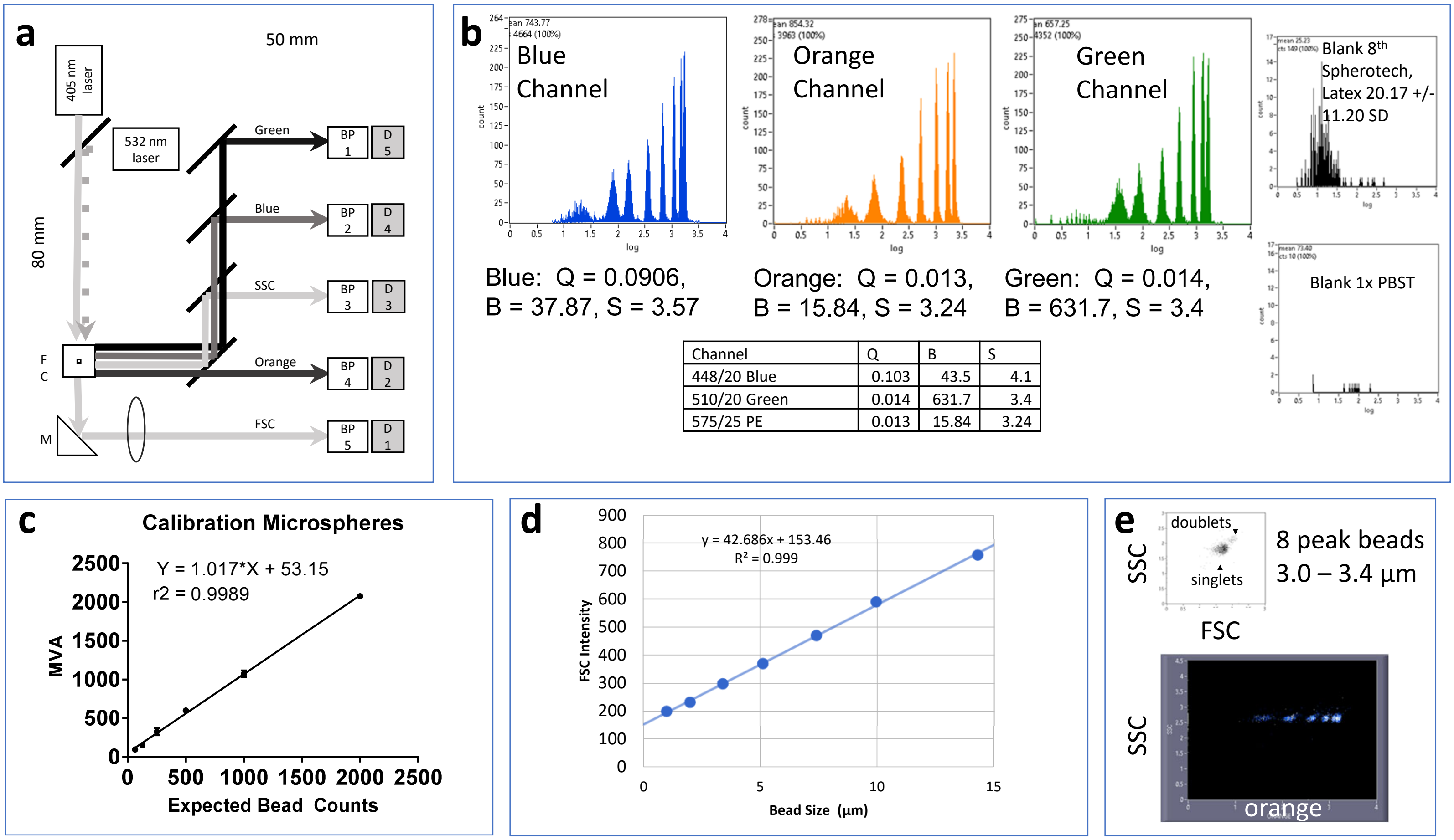


| Channel | Q | B | S |
|---|---|---|---|
| 448/20 Blue | 0.103 | 43.5 | 4.1 |
| 510/20 Green | 0.014 | 631.7 | 3.4 |
| 575/25 PE | 0.013 | 15.84 | 3.24 |

**Supplementary Fig. 2.** ***Optical cytometry module performance.*** **a**, Layout of the 50 mm x 80 mm optical block. The 405 nm and 532 nm lasers are focused onto the flow cell (FC). Orthogonal fluorescence light is split by dichroic filters and filtered by bandpass (BP) filters and captured by detectors (D1-5). The channels are green, blue, side scatter channel (SSC), orange, and forward scatter channel (FSC). **b**, Q (detection efficiency), B (background), and S (separation) analysis of the fluorescence channels. *Top right,* histogram for detection of blank autofluorescent beads is shown. *Bottom right,* run with 1x PBST negative control, showing confirmation that the 8$^{th}$ autofluorescent peak can be detected. **c**, The Molecules of Equivalent Cascade Blue (MECSB) is plotted versus 256 channels. **d**, FSC intensity is plotted versus bead size (µm). This size histogram for 1.0-1.4 µm beads in the SSC channel is displayed, showing resolution from noise. **e**, Scatterplots of FSC and SSC for 3.0-3.4 µm beads showing singlets and doublets. SSC plotted versus orange for calibration beads.

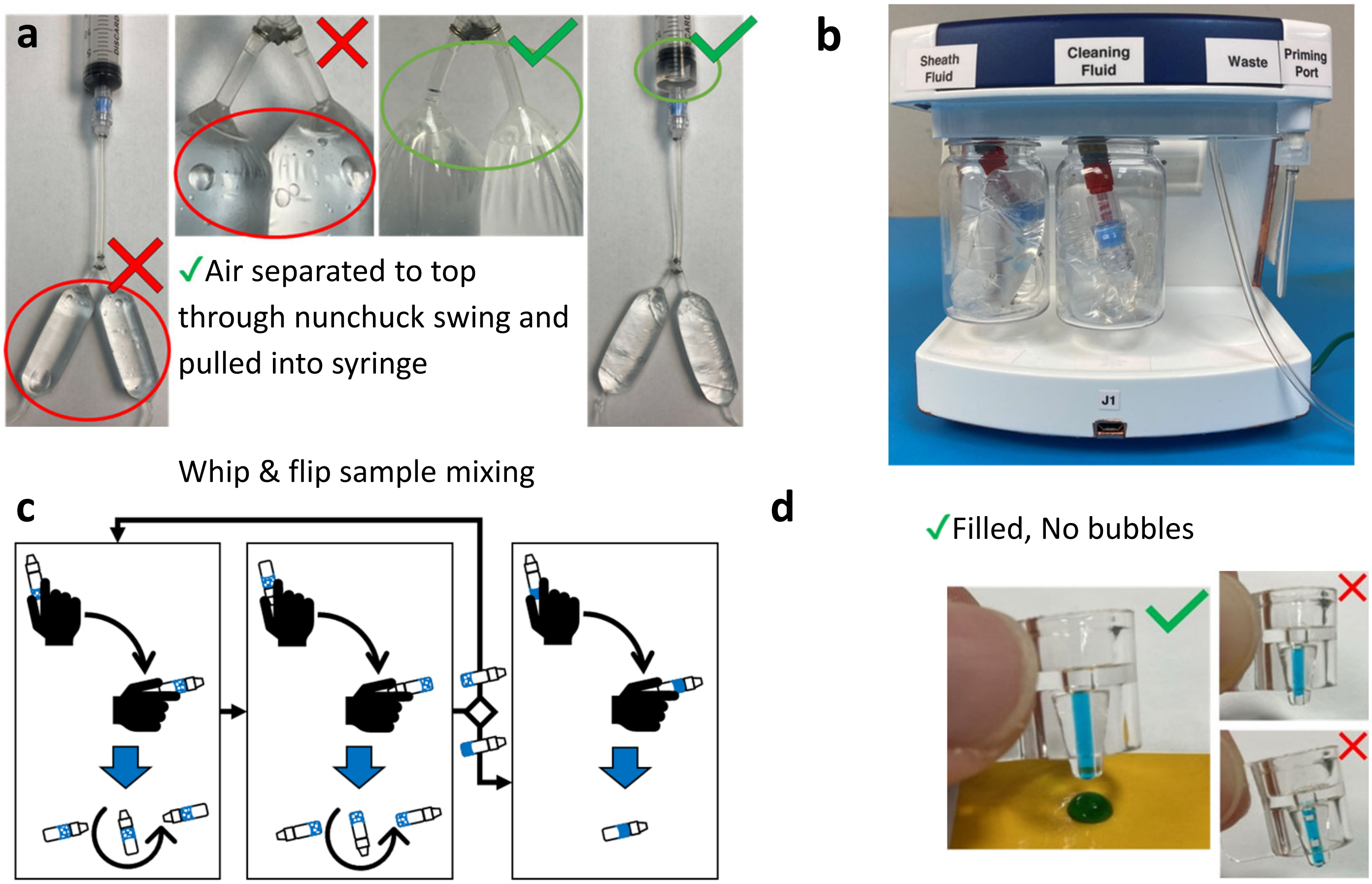


**Supplementary Fig. 3.** ***Techniques for bubble-free filling and loading in microgravity.*** **a**, *Left two images,* improperly filled fluid bag with air bubbles after a nunchuck swing, which creates a force vector to localize bubbles near the syringe injection area. *Right two images,* correctly filled fluid bag with no air bubbles. **b**, rHEALTH ONE with the correctly filled fluid bag inside the plastic bottles. **c**, *Left-to-right,* description of the whip and flip sample mixing where the sample was whipped and flipped with the cap pointing out, then with the cap pointing in, then the cap pointing out just prior to dispensing. **d**, *Left*, correctly filled sample consumable without any air bubbles. *Right two images*, two examples of improperly filled air bubbles, one with air bubbles at the top and another with air bubbles in the middle.

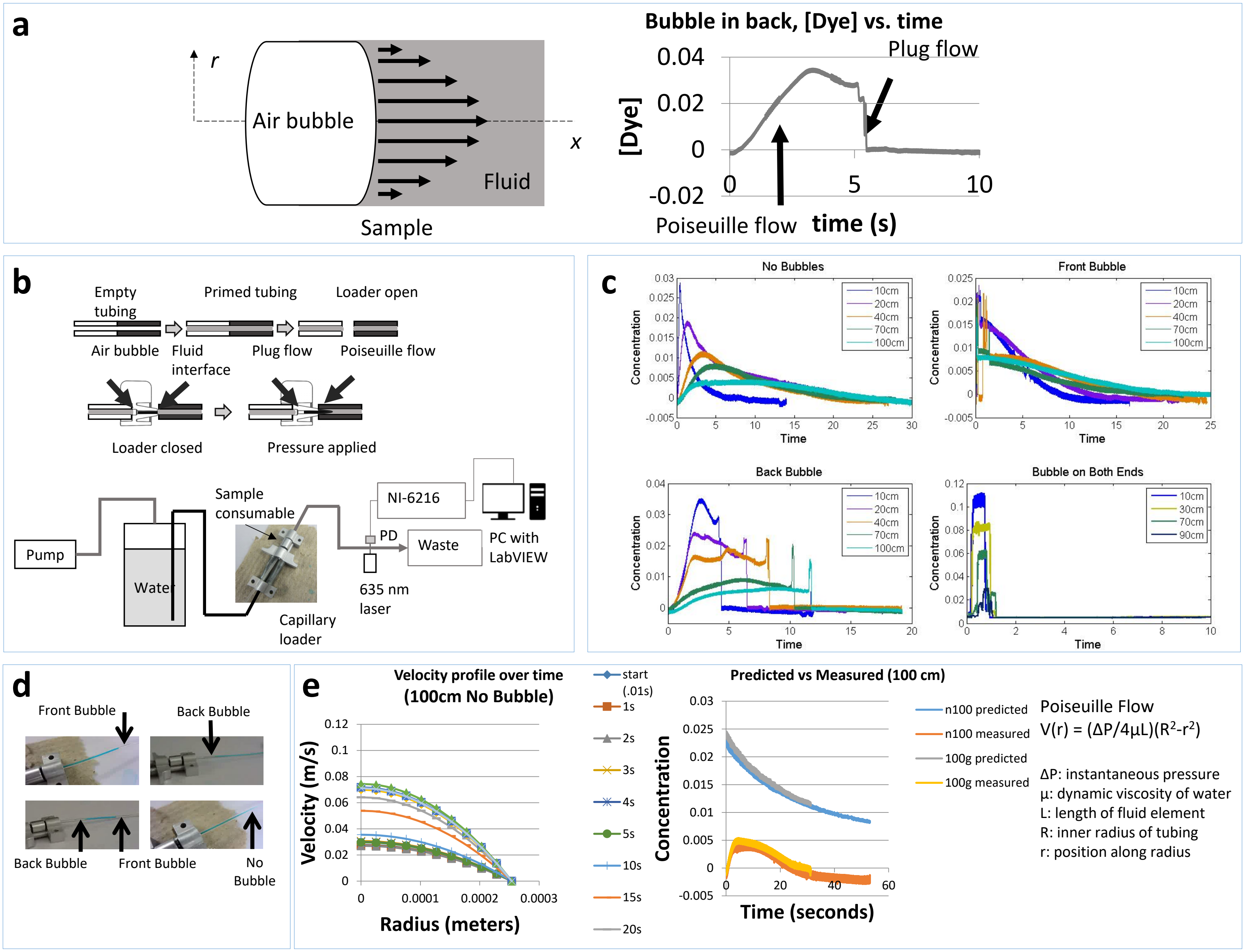


**Supplementary Fig. 4.** ***Sample loader characterization.*** **a**, Sample loading profile showing Poiseuille flow at the leading edge and plug flow in the back where the air bubble is. The graph shows relative blue dye concentration [dye] versus time of transit through the detector, showing the different sample profiles at the leading and trailing edges. **b**, Sample loading sequence, starting with empty tubing, which is primed with buffer. The interface is opened and the sample consumable is loaded into the system. Pressure is applied, resulting in a fluid interface at the leading edge and an air bubble at the trailing edge. The in-line loading approach allows for controlled sample loading with a fluid-fluid interface at the leading edge and an air-fluid interface at the trailing edge. The sample loading test apparatus is also shown, which was utilized to test various bubble loading cases. An air pump is utilized to pressurize a water container, which then pushes water through tubing through the sample loader. A 635 nm laser is utilized to measure the loading profiles, which are captured by a photodiode connected to an NI-6216 DAQ card attached to a computer running LabVIEW. **c**, Loading profiles of the four cases: (1) no bubbles, (2) front bubble, (3) back bubble, and (4) bubble on both ends. In all cases, tubing lengths from 10 to 100 cm, in increments of 10 cm, were tested. **d**, Various cases of sample loading testing with blue dye. The bubble can be at the front, back, both ends, or none on either side. **e**, The velocity profiles along the radium of 100 cm tubing, where r = 0 is the center axis of the tubing. The fastest velocity is found at the center of the tubing. The calculation can be utilized to predict the relative dilution of the sample at the leading edge of the sample. Poiseuille flow describes a parabolic velocity profile of a fluid which matches the observed data.

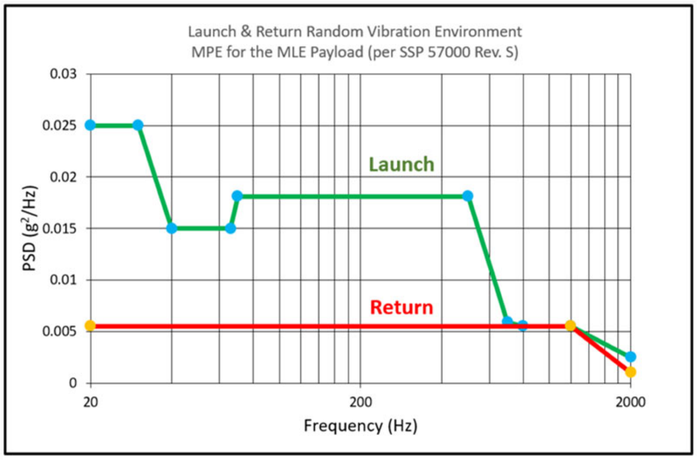


**Supplementary Fig. 5.** ***Vibration profiles utilized to qualify the rHEALTH ONE for flight per NASA's Pressurized Payloads Interface Requirements Document SSP 57000 Rev. S.*** Vibration frequency (Hz) plotted versus the Power Spectral Density (PSD) in $g^2$/Hz. The Maximum Permissible Exposure (MPE) is graphed for the Middeck Locker Equivalent (MLE). The launch is subject to higher PSD than the return.

## Spherotech RQC-30-5: Counting Methodology

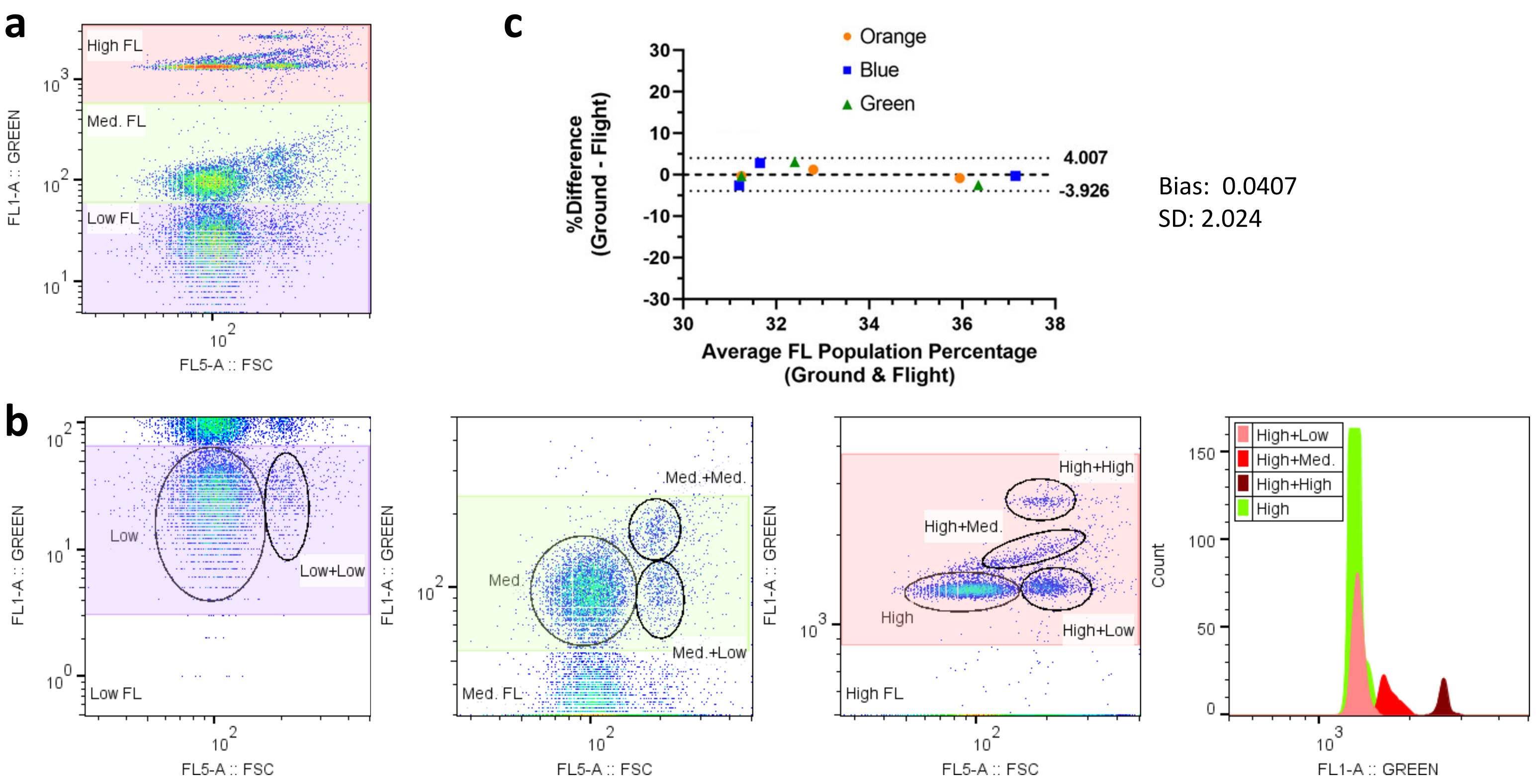


**Supplementary Fig. 6.** ***Coincident event counting methodology for Spherotech RQC-30-5 beads.*** **a**, FSC versus green scatterplot showing the three different low (Low), medium (Med), and high (High) fluorescent (FL) populations. The low events are boxed in purple, medium in green, and high in red. **b**, Zoom-in for each of the event boxes. *Left-to-right,* purple boxed low events showing singlet events (Low) and coincident events paired with other low events (Low + Low), green boxed medium events showing singlet medium events (Med) can pair with Med or Low events (Med + Low, Med + Med), red boxed high events showing singlet high events (High) and coincident pairing in all combinations (High + Low, High + Med, and High + High), and red boxed histogram view of only the high events with the green histogram as the singlet and the red histograms (in various shades) as doublets. **c**, The percentage of counted events for the three populations were compared using a Bland-Altman plot comparing the ground and flight percentage events. The x-axis shows the average percentage for flight and ground and the y-axis shows the % difference between the ground and flight.

Preflight Ground Data - Gallios

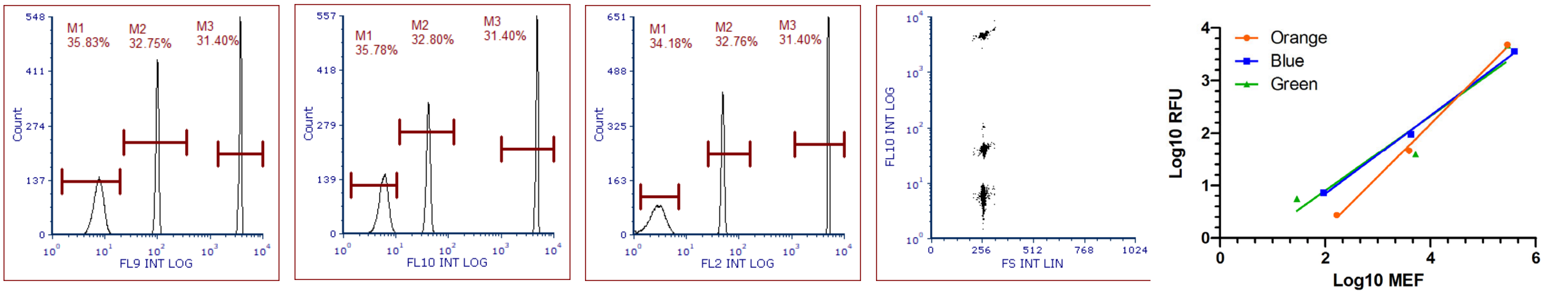


GALLIOS GROUND DATA

LMD 16435

Date: Oct 29, 2021

Flt reagents assayed PRE flight check

**Supplementary Fig. 7.** ***Benchmark preflight Gallios data for Spherotech RQC-30-5 beads.*** *Left-to-right,* blue (FL9) histogram with gates M1 – M3 calculating the bead percentages, green (FL10) histogram showing gates M1 – M3, orange (FL2), and FSC versus green scatterplot, and Molecules of Equivalent Fluorochrome (MEF) versus median $\log_{10}$ RFU for each population.

a

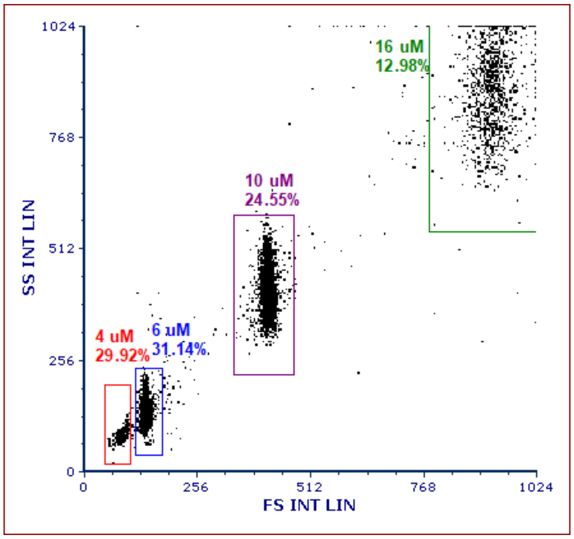


b

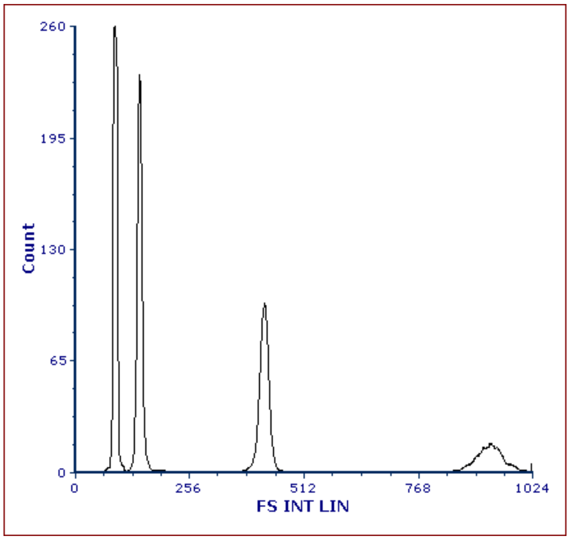


c

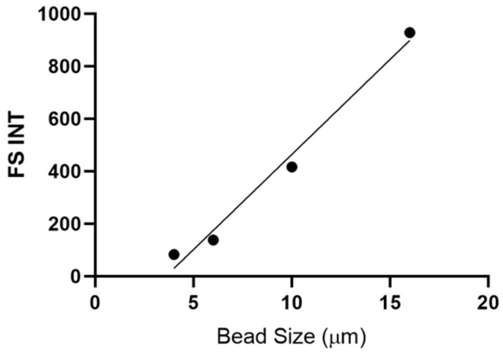


d

| Gate | # of Events | X Geometric Mean | Y Geometric Mean | % of gated cells (from plot) | % of all cells |
|---|---|---|---|---|---|
| None | 10125 | 202.74 | 189.55 | 100.00 | 100.00 |
| 4 uM | 3029 | 83.90 | 75.46 | 29.92 | 29.92 |
| 6 uM | 3153 | 138.93 | 128.90 | 31.14 | 31.14 |
| 10 uM | 2486 | 417.18 | 407.20 | 24.55 | 24.55 |
| 16 uM | 1314 | 928.59 | 881.55 | 12.98 | 12.98 |

GALLIOS GROUND DATA

LMD 16436

Date: Oct 29, 2021

PRE flight check of flt reagents

**Supplementary Fig. 8.** ***Benchmark preflight Gallios data for Spherotech PPS-6k beads.*** (A) FSC versus SSC scatterplot for the beads. Various color gates are utilized to identify each population. (B) FSC histogram of the four populations. (C) Bead diameter in μm versus the mean FSC intensity (FS INT). (D) Summary statistics for each of the gates shown in A.

| Features | Description |
|---|---|
| Product Name | rHEALTH ONE |
| Product Description | Compact small volume, high sensitivity biomarker analyzer |
| **General** | |
| Size | Height: 5.1” (13.0 cm)<br>Width: 5.3” (13.4 cm)<br>Depth: 7.0” (17.8 cm) |
| Weight | 3.3 lb (1.5 kg) |
| Operating environment | 15°C to 35°C |
| Power, data | USB 2.0 |
| Water supply | None required |
| Air supply | None required |
| Laser safety | Class I laser product |
| **Optics** | |
| Lasers | 405 nm violet (5 mW); 532 nm green (20 mW). Life expectancy > 5000 hrs for each laser |
| Detectors | Solid-state, high-gain photon counting detectors for all channels |
| Detection Channels | *405 nm laser*: Blue fluorescence (450 nm), Green fluorescence (510 nm). *532 nm laser*: Yellow fluorescence (575 nm), FSC, SSC. |
| **Fluidics** | |
| Sample volume | 5-10 µL |
| Sample flow rate | 2 – 10 µL/minute |
| Fluid reservoirs | Sheath, cleaner, and waste at 60 mL each |
| Sample analysis time | < 3 minutes |
| Throughput | 1000 events per second |
| Particle size | 2-60 µm |
| Sample concentration | $10^4$ to $10^7$ particles per mL |
| Sample loader | In-line, no dead-volume 10 µL plastic consumable |
| Cleaning | Automated |
| **Signal Processing and Analysis** | |
| Assay dynamic range | Five log decade range |
| Software | rHEALTH Viewer and Capture Software |
| Data format | All digital |
| Minimum computing requirements | 2.8-GHz processor, 2 GB RAM, 256 GB storage. |
| **Assay Particles Supported** | |
| Assays | Ultra-high sensitivity Nanostrips and Lumibeads, cell analysis, multiplexed microsphere assays. |

**Supplementary Table 1.** ***rHEALTH ONE base device specifications.*** The device, prior to payload development, for the NASA COTS program.

| Sample Letter for Flight | Control Solution P/N (Manufacturer) | Description | Feature Tested | Applicable Channel(s) | Feature in Raw Data |
|---|---|---|---|---|---|
| **A** | 01-1111-42 (Thermo Fisher)<br><br>85-0038-T100<br>75-0149-T100<br>50-0199-T100<br>(Tonbo Biosciences) | Antigen-capture OneComp eBeads stained with antibodies:<br>CD3 V500,<br>CD14 V450,<br>CD19 PE | Spectral Overlap | GREEN<br>BLUE<br>ORANGE<br>(Respectively to dyes) | Target: 1 distinct amplitude, peaks not repeated on other color channels |
| **B** | PPS-6K (Spherotech) | Particle Size Standard Kit –using 4, 6, 10, and 15µm | Particle size resolution | FSC | Target: 4 distinct amplitudes |
| **C** | RQC-30-5 (Spherotech) | Rainbow QC Calibration Particles, 3 peaks of intensity, all colors | Fluorescence resolution: sensitivity and linearity of the instrument | GREEN<br>BLUE<br>ORANGE | Target: 3 distinct amplitudes |
| **D** | A69184 (RUO) or A63492 (IVD) (Beckman Coulter) | Flow-Set Pro Fluorospheres uniform size and intensity | PMT Optical Precision, alignment | ALL | Target: Low CV |

**Supplementary Table 2.** ***Summary of samples tested.*** Detailed descriptions of the four samples (A-D).

| Frequency (Hz) | MPE PSD ($g^2/Hz$) |
|---|---|
| 20 | 0.0250 |
| 30 | 0.0250 |
| 40 | 0.0150 |
| 66 | 0.0150 |
| 70 | 0.0181 |
| 500 | 0.0181 |
| 700 | 0.0059 |
| 800 | 0.0055 |
| 1200 | 0.0055 |
| 2000 | 0.0025 |
| Grms | 4.063* |
| Duration (sec/axis) | 60 |

**Rounded to 4.07 in SSP 57000*

**Supplementary Table 3.** ***Simulated launch shock and vibration with frequency (Hz) and associated Maximum Permissible Exposure (MPE) $g^2$/Hz.*** The $G_{rms}$ is the root mean square gravity level for the various exposures. Based on NASA SSP 57000 Rev S.

| Frequency (Hz) | MPE PSD ($g^2/Hz$) |
|---|---|
| 20 | 0.0055 |
| 1200 | 0.0055 |
| 2000 | 0.0010 |
| Grms | 2.908** |
| Duration (sec/axis) | 60 |

***Rounded to 2.09 in SSP 57000*

**Supplementary Table 4.** ***Simulated return shock and vibration with frequency (Hz) and associated Maximum Permissible Exposure (MPE) $g^2$/Hz.*** Based on NASA SSP 57000 Rev S.

| Preflight Ground Data - Gallios<br>Trigger: FSC | | | |
|---|---|---|---|
| **Channel** | **Counts** | **Mean** | **%CV** |
| Blue: | 1682 | 232.71 | 12.61 |
| Green: | 1681 | 250.95 | 12.04 |
| Orange: | 1681 | 518.01 | 4.45 |
| SSC: | 1682 | 254.62 | 6.41 |
| FSC: | 1680 | 244.92 | 5.83 |
| Mean Channel Counts | 1681 | | |
| **Preflight Ground Data - rHEALTH ONE<br>Trigger: SSC** | | | |
| **Channel** | **Counts** | **Mean** | **%RCV** |
| Blue: | 5277 | 247.42 | 12.91 |
| Green: | 5263 | 113.20 | 19.02 |
| Orange: | 5257 | 520.80 | 6.33 |
| SSC: | 5380 | 557.20 | 12.26 |
| FSC: | 5390 | 78.54 | 26.89 |
| Mean Channel Counts | 5313 | | |
| **On-Orbit Flight Data - rHEALTH ONE<br>Trigger: SSC** | | | |
| **Channel** | **Counts** | **Mean** | **%RCV** |
| Blue: | 4072 | 245.88 | 11.77 |
| Green: | 4062 | 110.85 | 18.91 |
| Orange: | 4047 | 561.58 | 6.23 |
| SSC: | 4169 | 703.17 | 7.88 |
| FSC: | 4183 | 68.05 | 28.29 |
| Mean Channel Counts | 4107 | | |

**Supplementary Table 5.** ***Summary statistics for Flow-Set beads for ground (Gallios, rHEALTH ONE) and flight (rHEALTH ONE), as presented in Figure 5.*** The counts, %CV, and mean are shown for each channel and experiment.

| FILE | B1 MN | B1 SD | B1 CV | G1 MN | G1 SD | G1 CV | O1 MN | O1 SD | O1 CV | SSC1 MN | SSC1 SD | SSC1 CV | FSC1 MN | FSC1 SD | FSC1 CV |
|---|---|---|---|---|---|---|---|---|---|---|---|---|---|---|---|
| **GROUND** | | | | | | | | | | | | | | | |
| GMT343_SampleD4 | 247.66 | 30.99 | 12.52 | 113.13 | 20.98 | 18.54 | 520.70 | 31.95 | 6.14 | 556.85 | 68.35 | 12.28 | 78.49 | 20.12 | 25.64 |
| GMT343_PracticeD1 | 264.07 | 42.33 | 16.03 | 67.91 | 17.82 | 26.24 | 642.12 | 33.53 | 5.22 | 182.08 | 40.24 | 22.10 | 113.44 | 18.98 | 16.73 |
| GMT343_PracticeD2 | 314.73 | 41.99 | 13.34 | 93.08 | 18.70 | 20.09 | 673.99 | 31.72 | 4.71 | 203.99 | 33.15 | 16.25 | 112.68 | 19.63 | 17.42 |
| GMT343_SampleD1 | 268.01 | 32.75 | 12.22 | 118.20 | 22.61 | 19.13 | 541.75 | 39.55 | 7.30 | 522.38 | 81.87 | 15.67 | 40.83 | 13.04 | 34.38 |
| GMT343_SampleD2 | 273.79 | 30.50 | 11.14 | 127.54 | 21.25 | 16.66 | 565.24 | 33.71 | 5.96 | 589.43 | 70.62 | 11.98 | 90.52 | 21.18 | 23.40 |
| GMT343_SampleD3 | 247.67 | 29.61 | 11.95 | 115.34 | 20.74 | 17.98 | 503.52 | 31.56 | 6.27 | 541.20 | 62.51 | 11.55 | 80.98 | 19.78 | 24.42 |
| | | | | | | | | | | | | | | | |
| **FLIGHT** | | | | | | | | | | | | | | | |
| GMT136_Samplek1d1 | 245.92 | 28.22 | 11.47 | 111.08 | 20.50 | 18.45 | 561.59 | 34.84 | 6.20 | 702.88 | 59.38 | 8.45 | 67.93 | 18.45 | 27.16 |
| GMT133_Sampled1 | 234.89 | 36.43 | 15.51 | 62.45 | 16.46 | 26.36 | 634.88 | 32.62 | 5.14 | 285.92 | 82.99 | 29.02 | 67.72 | 16.49 | 24.36 |
| GMT133_Sampled2 | 285.74 | 54.66 | 19.13 | 78.07 | 22.28 | 28.54 | 665.92 | 30.19 | 4.53 | 236.00 | 41.52 | 17.59 | 62.49 | 16.32 | 26.12 |
| GMT133_Sampled3 | 340.15 | 44.82 | 13.18 | 95.87 | 20.26 | 21.13 | 677.16 | 37.62 | 5.56 | 242.03 | 36.92 | 15.25 | 54.71 | 14.73 | 26.93 |
| GMT136_Samplek1d2 | 279.64 | 31.34 | 11.21 | 121.08 | 21.78 | 17.99 | 656.94 | 41.87 | 6.37 | 756.56 | 62.45 | 8.25 | 79.03 | 20.23 | 25.60 |

**Supplementary Table 6.** ***Flow-Set run statistics for all runs.*** rHEALTH ground and flight run statistics, MN (mean), SD (standard deviation), and CV (coefficient of variation) for each channel, across all rHEALTH ONE experiments performed.

RQC-30-5 Spherotech Bead Analysis

| **Preflight Ground Data - Gallios** | | | | | | | | | | | | | | | |
|---|---|---|---|---|---|---|---|---|---|---|---|---|---|---|---|
| | **Blue** | | | | | **Green** | | | | | **Orange** | | | | |
| | **# of Events** | **Population %** | **Median** | **Geometric Mean** | **CV** | **# of Events** | **Population %** | **Median** | **Geometric Mean** | **CV** | **# of Events** | **Population %** | **Median** | **Geometric Mean** | **CV** |
| **Low FL** | 8232 | 35.83% | 7.23 | 7.14 | 20.87 | 8222 | 35.78% | 5.57 | 5.50 | 19.43 | 7854 | 34.18% | 2.74 | 2.71 | 30.76 |
| **Med. FL** | 7526 | 32.75% | 94.75 | 93.88 | 5.69 | 7536 | 32.80% | 39.60 | 39.35 | 10.47 | 7527 | 32.76% | 46.56 | 46.59 | 5.92 |
| **High FL** | 7215 | 31.40% | 3651.74 | 3579.75 | 4.70 | 7215 | 31.40% | 4655.53 | 4573.21 | 4.19 | 7215 | 31.40% | 4782.86 | 4766.75 | 1.69 |
| **Preflight Ground Data - rHEALTH ONE** | | | | | | | | | | | | | | | |
| **Trigger: SSC** | **Blue** | | | | | **Green** | | | | | **Orange** | | | | |
| | **# of Event** | **Population %** | **Median** | **Geometric Mean** | **RCV** | **# of Events** | **Population %** | **Median** | **Geometric Mean** | **RCV** | **# of Events** | **Population %** | **Median** | **Geometric Mean** | **RCV** |
| **Low FL** | 9081 | 37.1% | 31.98 | 30.59 | 37.46 | 8545 | 35.9% | 26.03 | 24.96 | 42.24 | 8396 | 35.8% | 35.98 | 34.55 | 38.94 |
| **Med. FL** | 7850 | 32.1% | 101.02 | 99.99 | 21.72 | 7837 | 32.9% | 96.05 | 95.38 | 20.32 | 7739 | 33.0% | 155.13 | 155.20 | 16.14 |
| **High FL** | 7523 | 30.8% | 1057.87 | 1068.84 | 3.69 | 7413 | 31.2% | 1313.68 | 1326.43 | 3.06 | 7307 | 31.2% | 1534.85 | 1542.39 | 2.95 |
| **On-Orbit Flight Data - rHEALTH ONE** | | | | | | | | | | | | | | | |
| **Trigger: SSC** | **Blue** | | | | | **Green** | | | | | **Orange** | | | | |
| | **# of Events** | **Population %** | **Median** | **Geometric Mean** | **RCV** | **# of Events** | **Population %** | **Median** | **Geometric Mean** | **RCV** | **# of Events** | **Population %** | **Median** | **Geometric Mean** | **RCV** |
| **Low FL** | 10568 | 37.2% | 35.98 | 34.95 | 31.94 | 10696 | 36.8% | 50.05 | 48.92 | 27.96 | 10180 | 36.1% | 60.99 | 59.44 | 28.66 |
| **Med. FL** | 8858 | 31.2% | 93.07 | 92.06 | 23.57 | 9253 | 31.9% | 122.07 | 121.18 | 19.64 | 9208 | 32.6% | 169.10 | 168.36 | 16.55 |
| **High FL** | 8964 | 31.6% | 954.31 | 963.59 | 5.06 | 9097 | 31.3% | 1310.92 | 1321.99 | 4.85 | 8836 | 31.3% | 1610.90 | 1623.06 | 2.85 |

**Supplementary Table 7.** ***Summary statistics for Spherotech RQC-30-5 beads, as presented in Figure 6.*** The summary statistics for each low, middle, high population for each color channel is summarized for the ground Gallios, ground rHEALTH ONE and flight rHEALTH ONE experiments.

| FILE | B1 MN | B1 SD | B1 CV | B2 MN | B2 SD | B2 CV | B3 MN | B3 SD | B3 CV |
|---|---|---|---|---|---|---|---|---|---|
| **GROUND** | | | | | | | | | |
| GMT342_PracticeC1 | 33.94 | 12.13 | 35.75 | 104.56 | 20.52 | 19.62 | 1056.10 | 37.45 | 3.55 |
| GMT342_PracticeC2 | 24.50 | 9.34 | 38.18 | 87.98 | 18.80 | 21.36 | 1047.57 | 38.50 | 3.68 |
| GMT343_SampleC1 | 83.55 | 23.67 | 28.32 | 215.61 | 39.25 | 18.21 | 1491.08 | 167.93 | 11.26 |
| GMT343_SampleC2 | 104.80 | 25.23 | 24.08 | 252.35 | 39.69 | 15.73 | 1695.52 | 170.87 | 10.30 |
| GMT343_SampleC3 | 117.24 | 33.35 | 28.45 | 281.85 | 42.05 | 14.92 | 1739.75 | 140.17 | 8.06 |
| | | | | | | | | | |
| **FLIGHT** | | | | | | | | | |
| GMT136_Samplek1c1 | 35.40 | 9.93 | 28.04 | 94.45 | 19.38 | 20.52 | 950.94 | 40.67 | 4.28 |
| GMT133_practicec2 | 88.49 | 17.29 | 19.54 | 157.23 | 20.00 | 12.72 | 1075.59 | 42.68 | 3.97 |
| GMT136_Samplec1 | 74.58 | 17.86 | 23.95 | 192.41 | 31.46 | 16.36 | 1321.56 | 130.41 | 9.87 |
| GMT136_Samplec2 | 119.07 | 22.62 | 19.00 | 262.99 | 33.73 | 12.82 | 1600.32 | 136.04 | 8.50 |
| GMT136_Samplec3 | 110.76 | 18.18 | 16.41 | 198.46 | 35.12 | 17.70 | 1511.13 | 143.77 | 9.51 |
| GMT136_Samplec4 | 89.44 | 17.22 | 19.25 | 172.35 | 46.71 | 27.10 | 1624.92 | 146.92 | 9.05 |
| GMT136_Samplec5 | 46.13 | 15.69 | 34.01 | 124.21 | 16.23 | 13.07 | 1086.08 | 78.52 | 7.23 |
| GMT136_Samplec7 | 47.47 | 14.32 | 30.17 | 110.70 | 20.21 | 18.26 | 1057.26 | 42.73 | 4.04 |

| FILE | G1 MN | G1 SD | G1 CV | G2 MN | G2 SD | G2 CV | G3 MN | G3 SD | G3 CV |
|---|---|---|---|---|---|---|---|---|---|
| **GROUND** | | | | | | | | | |
| GMT342_PracticeC1 | 27.88 | 10.71 | 38.42 | 98.15 | 18.41 | 18.75 | 1310.45 | 35.07 | 2.68 |
| GMT342_PracticeC2 | 17.87 | 7.93 | 44.39 | 77.80 | 16.21 | 20.83 | 1267.24 | 41.40 | 3.27 |
| GMT343_SampleC1 | 19.58 | 8.56 | 43.73 | 94.68 | 21.63 | 22.84 | 1422.99 | 152.31 | 10.70 |
| GMT343_SampleC2 | 24.16 | 8.90 | 36.83 | 101.75 | 21.86 | 21.49 | 1508.19 | 111.42 | 7.39 |
| GMT343_SampleC3 | 23.55 | 8.88 | 37.68 | 100.30 | 20.27 | 20.21 | 1521.95 | 102.39 | 6.73 |
| | | | | | | | | | |
| **FLIGHT** | | | | | | | | | |
| GMT136_Samplek1c1 | 50.02 | 13.50 | 26.99 | 123.95 | 19.79 | 15.97 | 1304.7 | 53.12 | 4.07 |
| GMT133_practicec2 | 110.77 | 15.75 | 14.22 | 176.14 | 19.59 | 11.12 | 1338.31 | 51.77 | 3.87 |
| GMT136_Samplec1 | 74.39 | 18.22 | 24.49 | 158.91 | 14.26 | 8.97 | 1359.26 | 108.83 | 8.01 |
| GMT136_Samplec2 | 110.70 | 18.43 | 16.65 | 199.81 | 20.87 | 10.44 | 1600.57 | 84.01 | 5.25 |
| GMT136_Samplec3 | 88.65 | 16.66 | 18.79 | 166.78 | 26.69 | 16.00 | 1500.20 | 105.28 | 7.02 |
| GMT136_Samplec4 | 78.92 | 15.08 | 19.11 | 142.45 | 28.22 | 19.81 | 1569.81 | 124.24 | 7.91 |
| GMT136_Samplec5 | 79.38 | 18.03 | 22.72 | 172.91 | 23.42 | 13.54 | 1499.39 | 109.36 | 7.29 |
| GMT136_Samplec7 | 69.28 | 16.64 | 24.02 | 148.7 | 22.74 | 15.29 | 1459.11 | 59.88 | 4.10 |

| FILE | O1 MN | O1 SD | O1 CV | O2 MN | O2 SD | O2 CV | O3 MN | O3 SD | O3 CV |
|---|---|---|---|---|---|---|---|---|---|
| **GROUND** | | | | | | | | | |
| GMT342_PracticeC1 | 37.34 | 13.78 | 36.90 | 156.22 | 24.81 | 15.88 | 1536.14 | 45.40 | 2.96 |
| GMT342_PracticeC2 | 27.99 | 11.35 | 40.54 | 151.70 | 22.13 | 14.59 | 1531.39 | 41.11 | 2.68 |
| GMT343_SampleC1 | 75.46 | 22.78 | 30.19 | 260.55 | 32.23 | 12.37 | 2042.49 | 108.15 | 5.30 |
| GMT343_SampleC2 | 83.23 | 28.52 | 34.26 | 261.30 | 42.89 | 16.41 | 2182.69 | 122.06 | 5.59 |
| GMT343_SampleC3 | 112.59 | 33.05 | 29.36 | 300.83 | 42.88 | 14.25 | 2283.48 | 107.70 | 4.72 |
| | | | | | | | | | |
| **FLIGHT** | | | | | | | | | |
| GMT136_Samplek1c1 | 62.46 | 15.28 | 24.46 | 169.41 | 26.75 | 15.79 | 1614.53 | 45.74 | 2.83 |
| GMT133_practicec2 | 57.85 | 17.81 | 30.79 | 165.39 | 23.46 | 14.18 | 1741.81 | 48.58 | 2.79 |
| GMT136_Samplec1 | 88.93 | 20.87 | 23.47 | 240.43 | 27.25 | 11.33 | 1817.37 | 91.13 | 5.01 |
| GMT136_Samplec2 | 121.02 | 21.47 | 17.74 | 290.97 | 27.99 | 9.62 | 2069.50 | 75.78 | 3.66 |
| GMT136_Samplec3 | 127.79 | 25.30 | 19.79 | 275.90 | 37.10 | 13.45 | 1961.00 | 76.01 | 3.88 |
| GMT136_Samplec4 | 123.47 | 24.30 | 19.68 | 316.24 | 59.51 | 18.82 | 2340.78 | 247.29 | 10.56 |
| GMT136_Samplec5 | 67.14 | 16.43 | 24.48 | 190.63 | 31.80 | 16.68 | 1813.61 | 67.40 | 3.72 |
| GMT136_Samplec7 | 84.97 | 23.27 | 27.39 | 203.66 | 29.77 | 14.62 | 1807.50 | 51.04 | 2.82 |

**Supplementary Table 8.** ***Spherotech RQC-30-5 run statistics for blue, green, and orange channels for all runs.*** rHEALTH ground and flight run statistics, MN (mean), SD (standard deviation), and CV (coefficient of variation) for each of the three blue (B1-3), green (G1-G3), and orange populations (O1-3), across all rHEALTH ONE experiments performed.

| Preflight Ground Data - rHEALTH ONE | | | | | |
|---|---|---|---|---|---|
| **Bead Size** | **FSC** | | | | |
| | **# of Events** | **Population %** | **Median** | **Geometric Mean** | **RCV** |
| **4µm** | 802 | 18.45 | 41.02 | 39.14 | 78.01 |
| **6µm** | 872 | 20.06 | 81.05 | 78.80 | 26.52 |
| **10µm** | 942 | 21.66 | 272.08 | 268.20 | 16.49 |
| **15µm** | 1732 | 39.83 | 474.03 | 473.58 | 6.37 |
| **On-Orbit Flight Data - rHEALTH ONE** | | | | | |
| **Bead Size** | **FSC** | | | | |
| | **# of Events** | **Population %** | **Median** | **Geometric Mean** | **RCV** |
| **4µm** | 531 | 12.46 | 20.01 | 23.72 | 107.49 |
| **6µm** | 961 | 22.55 | 76.95 | 74.32 | 27.91 |
| **10µm** | 948 | 22.25 | 272.08 | 266.21 | 15.43 |
| **15µm** | 1821 | 42.74 | 411.73 | 406.00 | 10.41 |

**Supplementary Table 9.** ***Summary statistics for Spherotech PPS-6K beads, as presented in Figure 7.*** rHEALTH ground and flight summary statistics for each of the four gated populations (4, 6, 10, and 15 µm diameter beads).

| FILE | FS1 MN | FS1 SD | FS1 CV | FS2 MN | FS2 SD | FS2 CV | FS3 MN | FS3 SD | FS3 CV | FS4 MN | FS4 SD | FS4 CV |
|---|---|---|---|---|---|---|---|---|---|---|---|---|
| **GROUND** | | | | | | | | | | | | |
| GMT343_SampleB1 | 50.65 | 32.45 | 64.09 | 82.05 | 20.19 | 24.61 | 271.34 | 40.20 | 14.81 | 474.10 | 29.26 | 6.17 |
| GMT342_SampleB1 | 40.84 | 32.53 | 79.66 | 71.37 | 18.22 | 25.53 | 232.54 | 34.91 | 15.01 | 434.55 | 38.33 | 8.82 |
| GMT342_SampleB2 | 56.85 | 36.08 | 63.46 | 81.61 | 20.33 | 24.91 | 264.03 | 34.78 | 13.17 | 474.33 | 22.73 | 4.79 |
| GMT342_SampleB4 | 53.12 | 38.14 | 71.81 | 54.63 | 20.63 | 37.75 | 234.77 | 46.07 | 19.62 | 640.05 | 108.35 | 16.93 |
| GMT342_SampleB5 | 18.67 | 6.32 | 33.87 | 56.20 | 15.43 | 27.45 | 175.77 | 52.37 | 29.80 | 494.45 | 75.84 | 15.34 |
| GMT343_SampleB2 | 53.59 | 38.05 | 71.00 | 75.38 | 21.66 | 28.73 | 261.51 | 42.04 | 16.08 | 478.22 | 31.37 | 6.56 |
| | | | | | | | | | | | | |
| **FLIGHT** | | | | | | | | | | | | |
| GMT136_Samplek1b1 | 31.21 | 23.97 | 76.79 | 76.52 | 17.82 | 23.29 | 271.68 | 36.17 | 13.31 | 408.77 | 39.70 | 9.71 |
| GMT136_Sampleb1 | 56.64 | 33.93 | 59.91 | 91.46 | 16.73 | 18.29 | 303.60 | 24.09 | 7.94 | 387.26 | 62.34 | 16.10 |
| GMT136_Sampleb2 | 38.96 | 29.37 | 75.38 | 97.12 | 14.54 | 14.97 | 321.06 | 21.17 | 6.60 | 398.25 | 59.54 | 14.95 |
| GMT136_Sampleb3 | 37.55 | 31.25 | 83.23 | 100.80 | 13.99 | 13.88 | 329.68 | 26.89 | 8.16 | 411.25 | 61.32 | 14.91 |
| GMT136_Sampleb4 | 24.26 | 18.61 | 76.72 | 108.00 | 17.07 | 15.80 | 329.61 | 29.69 | 9.01 | 422.37 | 60.48 | 14.32 |
| GMT136_Samplek1b2 | 30.79 | 35.72 | 116.03 | 161.34 | 54.60 | 33.84 | 606.82 | 119.6 | 19.71 | 1063.54 | 258.88 | 24.34 |

**Supplementary Table 10.** ***Spherotech PPS-6K run statistics for all runs.*** rHEALTH ONE ground and flight run statistics, MN (mean), SD (standard deviation), and CV (coefficient of variation) for each of the four gated populations (FS1, FS2, FS3, and FS4), across all experiments performed.

| Preflight Ground Data - Gallios | | | | | |
|---|---|---|---|---|---|
| **FL9 vs FL10** | **Percentages** | **FL2 vs FL9** | **Percentages** | **FL2 vs FL10** | **Percentages** |
| TL (FL10+ , FL9-) | 13.15% | TL (FL9+ , FL2-) | 13.43% | TL (FL10+ , FL2-) | 13.07% |
| TR (FL10+ , FL9+) | 0% | TR (FL9+ , FL2+) | 0.15% | TR (FL10+ , FL2+) | 0.08% |
| LL (FL9- , FL10-) | 73.28% | LL (FL2- , FL9-) | 70.92% | LL (FL2- , FL10-) | 71.37% |
| LR (FL9+ , FL10-) | 13.57% | LR (FL2+ ,FL9-) | 15.51% | LR (FL2+ , FL10-) | 15.48% |
| **Preflight Ground Data - rHEALTH ONE** | | | | | |
| **Blue vs Green** | **Percentages & Counts** | **Orange vs Blue** | **Percentages & Counts** | **Orange vs Green** | **Percentages & Counts** |
| TL (GR+ , BL-) | 12.52% (956) | TL (BL+ , OR-) | 13.12% (1002) | TL (GR+ , OR-) | 12.67% (970) |
| TR (GR+ , BL+) | 0.75% (57) | TR (BL+ , OR+) | 0.75% (57) | TR (GR+ , OR+) | 0.60% (46)LL |
| LL (BL- , GR- ) | 73.99% (5649) | LL (OR- , BL-) | 70.33% (5370) | LL (OR- , GR- ) | 70.70% (5395) |
| LR (BL+ , GR-) | 12.74% (973) | LR (OR+ , BL-) | 15.80% (1206) | LR (OR+ , GR-) | 16.03% (1224) |
| **On-Orbit Flight Data - rHEALTH ONE** | | | | | |
| **Blue vs Green** | **Percentages & Counts** | **Orange vs Blue** | **Percentages & Counts** | **Orange vs Green** | **Percentages & Counts** |
| TL | 11.02% (795) | TL | 0.53% (38) | TL | 0.29% (21) |
| TR | 2.37% (171) | TR | 15.01% (1083) | TR | 13.25% (956) |
| LL | 75.32% (5433) | LL | 56.91% (4105) | LL | 57.06% (4116) |
| LR | 11.29% (814) | LR | 27.55% (1987) | LR | 29.39% (2120) |
| **Postflight - Gallios** | | | | | |
| **FL9 vs FL10** | **Percentages** | **FL2 vs FL9** | **Percentages** | **FL2 vs FL10** | **Percentages** |
| TL | 12.58% | TL | 13.19% | TL | 12.33% |
| TR | 0% | TR | 0% | TR | 0% |
| LL | 74.22% | LL | 71.75% | LL | 72.75% |
| LR | 13.19% | LR | 15.05% | LR | 14.93% |

**Supplementary Table 11.** ***Summary statistics for OneComp eBeads with CD19 PE, CD14 V450, and CD3 V500-labeled antibodies, as presented in Figure 8.*** The data for the quadrant analysis for preflight ground Gallios, ground rHEALTH ONE, flight rHEALTH ONE, and postflight Gallios are shown. The quadrant gates for the Gallios are denoted by TL (top left), TR (top right), LL (lower left), and LR (lower right). The Gallios gates postflight were moved to match the percentages in each quadrant.

| FILE | B1 MN | B1 SD | B1 CV | G1 MN | G1 SD | G1 CV | O1 MN | O1 SD | O1 CV | O2 MN | O2 SD | O2 CV | SSC1 MN | SSC1 SD | SSC1 CV | FSC1 MN | FSC1 SD | FSC1 CV |
|---|---|---|---|---|---|---|---|---|---|---|---|---|---|---|---|---|---|---|
| **GROUND** | | | | | | | | | | | | | | | | | | |
| GMT342_SampleA1 | 73.74 | 27.82 | 37.73 | 81.79 | 24.51 | 29.96 | NA | NA | NA | 915.16 | 100.46 | 10.98 | 571.07 | 183.46 | 32.12 | 333.19 | 26.61 | 7.99 |
| GMT342_SampleA2 | 87.97 | 24.07 | 27.36 | 79.32 | 22.72 | 28.64 | NA | NA | NA | 953.76 | 106.23 | 11.14 | 497.07 | 168.17 | 33.83 | 344.21 | 26.27 | 7.63 |
| GMT342_SampleA3 | 92.48 | 27.80 | 30.06 | 78.70 | 23.72 | 30.13 | NA | NA | NA | 1007.21 | 107.27 | 10.65 | 436.77 | 155.42 | 35.58 | 361.47 | 24.45 | 6.76 |
| | | | | | | | | | | | | | | | | | | |
| **FLIGHT** | | | | | | | | | | | | | | | | | | |
| GMT136_Samplea2 | 53.07 | 17.47 | 32.91 | 75.77 | 23.52 | 31.04 | 243.35 | 45.98 | 18.90 | 751.37 | 82.55 | 10.99 | 769.82 | 194.75 | 25.30 | 234.44 | 34.54 | 14.73 |
| GMT133_Samplea1 | 54.16 | 23.35 | 43.11 | 52.41 | 18.18 | 34.69 | 340.54 | 50.32 | 14.78 | 954.12 | 95.35 | 9.99 | 672.97 | 225.96 | 33.58 | 258.96 | 20.40 | 7.90 |
| GMT133_Samplea2 | 60.70 | 22.76 | 37.50 | 52.71 | 16.80 | 31.88 | 344.33 | 59.61 | 17.31 | 989.24 | 103.46 | 10.46 | 612.72 | 222.60 | 36.33 | 272.86 | 21.11 | 7.74 |
| GMT133_Samplea3 | 51.17 | 21.09 | 41.22 | 51.31 | 14.81 | 28.87 | 356.11 | 56.08 | 15.75 | 1015.67 | 105.42 | 10.38 | 558.64 | 218.48 | 39.11 | 297.98 | 22.68 | 7.61 |
| GMT136_Samplea1 | 41.94 | 14.46 | 34.47 | 62.12 | 22.32 | 35.93 | 223.97 | 43.46 | 19.41 | 735.32 | 84.98 | 11.56 | 811.29 | 199.81 | 24.63 | 238.89 | 37.18 | 15.56 |
| GMT136_Samplek1a1 | 50.54 | 18.66 | 36.92 | 73.66 | 25.40 | 34.47 | 232.18 | 58.82 | 25.34 | 791.08 | 114.22 | 14.44 | 904.09 | 217.94 | 24.11 | 280.17 | 43.88 | 15.66 |
| GMT136_Samplek1a2 | 47.00 | 16.02 | 34.08 | 66.62 | 22.95 | 34.45 | 198.69 | 49.91 | 25.12 | 679.92 | 98.31 | 14.46 | 768.72 | 189.06 | 24.59 | 241.63 | 35.43 | 14.66 |

**Supplementary Table 12.** ***OneComp eBeads run statistics for all runs.*** rHEALTH ground and flight run statistics, MN (mean), SD (standard deviation), and CV (coefficient of variation) for each gated population (B1, G1, O1, O2, SSC1 and FSC1), across all experiments performed for the rHEALTH ONE.

| Run Summary | | | | | |
|---|---|---|---|---|---|
| **On-orbit run summary** | **Sample A** | **Sample B** | **Sample C** | **Sample D** | **Total** |
| **# planned runs** | 3 | 3 | 5[1] | 3 | 14[2] |
| **# runs performed** | 7 (233%) | 6 (200%) | 10 (200%) | 5 (166%) | 28[2] (200%) |
| **Runs yielding data** | 7 (233%) | 6 (200%) | 8[3] (160%) | 5 (166%) | 26 (186%) |
| **Mean run duration** | | | | | 143.10s |
| **Mean raw data per run** | | | | | 71,550,000 |

[1]Includes 2 practice runs
[2]Excludes blank runs to test instrument fluidics
[3]1 run had no peaks, 1 run was incomplete

**Supplementary Table 13.** ***Summary of on-orbit runs.*** The total number of runs, including which runs yielded usable data, average run duration, and data points collected are shown. The (%) is the percentage of the planned runs.

| TEST MATRIX | |
|---|---|
| **OPS SESSION 1** | **OPS SESSION 2** |
| Blank Run | Blank Run |
| Practice C1 | Practice C1 (or D1*) |
| Practice C2 | Practice C2 (or D2*) |
| Sample A1 (OneComp eBeads) | Sample B1 (Spherotech PPS-6K) |
| Sample A2 | Sample B2 |
| Sample A3 | Sample B3 |
| Sample D1 (Flow-Set Pro) | Sample C1 (Spherotech RQC-30-5) |
| Sample D2 | Sample C2 |
| Sample D3 | Sample C3 |
| No time: At least 1 good run each | Replace C with reruns |
| Extra time: run all 4 samples | Rerun samples as needed |
| * C or D shows up best to check the analyzer. Choose based on data needed. | |
| **END Session 1** | **END Session 2** |

**Supplementary Table 14.** ***Original test sequence for Ops Session 1 and Ops Session 2.*** Planned test matrix for the two day operational sessions. Blank and practice runs start each session and then two of the four test samples were planned for each day.

| MISSION SUCCESS | | | | |
|---|---|---|---|---|
| **CRITERIA** | | **MINIMAL** | **SUBSTANTIAL** | **COMPLETE** |
| Number of Channels with Signal: | Scatter channel | ≥ 1 | 2 | 2 |
| | Color channel | ≥ 1 | 3 | 3 |
| Number of Test Points: | A | N/A | ≥ 1 | ≥ 3 |
| | B | ≥ 1 | ≥ 1 | ≥ 3 |
| | C | ≥ 1 | ≥ 1 | ≥ 3 |
| | D | ≥ 1 | ≥ 1 | ≥ 3 |
| Hydrodynamic Flow Focusing: | | Present | Present | Present |
| Post-Flight Analysis | A | N/A | 1 distinct population per channel | Comparable to Ground Data<br><br>(e.g. Number of peak, CV for optical precision, degree of spectral overlap) |
| | B | | ≥ 1 population on a scatter channel | |
| | C | | 1 distinct population per scatter channel, AND ≥ 1 population per color channel | |
| | D | | 1 distinct population per channel | |
| Data Collection per Sample: | | ≥ 20 events | ≥ 1000 events | ≥ 3 minutes OR full sample run (to end bubble) with 5μL worth of data (~1 minute of constant events) |

**Supplementary Table 15.** ***Predetermined success metrics for the experiments.*** NASA-determined metrics for minimal, substantial, and complete success for the experiments.

| rHEALTH Fluids and Power Consumption | | |
|---|---|---|
| | **Startup Prime Sequence** | **Sample Run + Ending Prime** |
| **Sheath** | 2.44 mL | 1.13 mL |
| **Clean** | 0.55 mL | 0.02 mL |
| **Waste** | 2.99 mL | 1.15 mL |
| **Power Consumption** | 0.147 Wh (528 J over 180 seconds, 2.93W) | 0.156 Wh (563 J over (192 seconds, 2.93W) |

**Supplementary Table 16.** ***rHEALTH fluids and power consumption.*** The system requires a startup full prime at the beginning of each experimental day/session. Afterwards, multiple individual sample runs (consuming less fluid) are performed. The power consumption is also shown.
