# Supplementary material for: Single drop cytometry onboard the International Space Station
Source: Nat Commun. 2024 Mar 25;15:2634. doi: 10.1038/s41467-024-46483-6 (PMC10963801; doi:10.1038/s41467-024-46483-6)
Supplement: Supplementary file 3 — Reporting Summary [file 41467_2024_46483_MOESM3_ESM.pdf]

nature portfolio

Corresponding author(s): *Double-anonymous peer review submissions: write DAPR and your manuscript number here instead of author names.*

Last updated by author(s): *YYYY-MM-DD*

Reporting Summary

Nature Portfolio wishes to improve the reproducibility of the work that we publish. This form provides structure for consistency and transparency in reporting. For further information on Nature Portfolio policies, see our Editorial Policies and the Editorial Policy Checklist.

Statistics

For all statistical analyses, confirm that the following items are present in the figure legend, table legend, main text, or Methods section.

n/a	Confirmed	
☐	☒	The exact sample size (*n*) for each experimental group/condition, given as a discrete number and unit of measurement
☐	☒	A statement on whether measurements were taken from distinct samples or whether the same sample was measured repeatedly
☒	☐	The statistical test(s) used AND whether they are one- or two-sided<br>*Only common tests should be described solely by name; describe more complex techniques in the Methods section.*
☒	☐	A description of all covariates tested
☐	☒	A description of any assumptions or corrections, such as tests of normality and adjustment for multiple comparisons
☐	☒	A full description of the statistical parameters including central tendency (e.g. means) or other basic estimates (e.g. regression coefficient) AND variation (e.g. standard deviation) or associated estimates of uncertainty (e.g. confidence intervals)
☒	☐	For null hypothesis testing, the test statistic (e.g. *F*, *t*, *r*) with confidence intervals, effect sizes, degrees of freedom and *P* value noted<br>*Give P values as exact values whenever suitable.*
☒	☐	For Bayesian analysis, information on the choice of priors and Markov chain Monte Carlo settings
☒	☐	For hierarchical and complex designs, identification of the appropriate level for tests and full reporting of outcomes
☒	☐	Estimates of effect sizes (e.g. Cohen's *d*, Pearson's *r*), indicating how they were calculated

Our web collection on statistics for biologists contains articles on many of the points above.

Software and code

Policy information about availability of computer code

Data collection	rHEALTH Capture 59e4, commercially available
Data analysis	rHEALTH Viewer v26.2f_exporthistogram, GraphPad Prism 9.5.1, FlowJo v10.8.1, Microsoft 365 Excel, Google Sheets, all commercially available

For manuscripts utilizing custom algorithms or software that are central to the research but not yet described in published literature, software must be made available to editors and reviewers. We strongly encourage code deposition in a community repository (e.g. GitHub). See the Nature Portfolio guidelines for submitting code & software for further information.

Data

Policy information about availability of data

All manuscripts must include a data availability statement. This statement should provide the following information, where applicable:

- Accession codes, unique identifiers, or web links for publicly available datasets
- A description of any restrictions on data availability
- For clinical datasets or third party data, please ensure that the statement adheres to our policy

Provide your data availability statement here.

Research involving human participants, their data, or biological material

Policy information about studies with human participants or human data. See also policy information about sex, gender (identity/presentation), and sexual orientation and race, ethnicity and racism.

Reporting on sex and gender	N/A
Reporting on race, ethnicity, or other socially relevant groupings	N/A
Population characteristics	N/A
Recruitment	N/A
Ethics oversight	N/A

Note that full information on the approval of the study protocol must also be provided in the manuscript.

Field-specific reporting

Please select the one below that is the best fit for your research. If you are not sure, read the appropriate sections before making your selection.

☒ Life sciences ☐ Behavioural & social sciences ☐ Ecological, evolutionary & environmental sciences

For a reference copy of the document with all sections, see nature.com/documents/nr-reporting-summary-flat.pdf

Life sciences study design

All studies must disclose on these points even when the disclosure is negative.

Sample size	28 samples total, minimum N = 3 (triplicates) per A-D sample. Sampling based on astronaut time availability and minimum triplicate repeats.
Data exclusions	2 runs did not yield data or incomplete data. One was a practice run (pre-established exclusion), another ran out of sheath (unanticipated exclusion).
Replication	Replicated results for samples are reported in tables. Minimum triplicate sampling for each of A-D samples.
Randomization	Samples were run in a predetermined order. This was not a grouped study that required randomization.
Blinding	The authors at DMI and rHEALTH were blinded to the samples utilized in the experiment.

Reporting for specific materials, systems and methods

We require information from authors about some types of materials, experimental systems and methods used in many studies. Here, indicate whether each material, system or method listed is relevant to your study. If you are not sure if a list item applies to your research, read the appropriate section before selecting a response.

Materials & experimental systems

n/a	Involved in the study	
☐	☒	Antibodies
☒	☐	Eukaryotic cell lines
☒	☐	Palaeontology and archaeology
☒	☐	Animals and other organisms
☒	☐	Clinical data
☒	☐	Dual use research of concern
☒	☐	Plants

Methods

n/a	Involved in the study	
☒	☐	ChIP-seq
☐	☒	Flow cytometry
☒	☐	MRI-based neuroimaging

Antibodies

Antibodies used	CD19-PE Tonbo Biosciences P/N 50-0199-Tl00 Lot C0199110320503, CD 14 V450 Tonbo Biosciences P/N 75-0149 Tl00 Lot C0149092019753, CD3 VS00 Tonbo Biosciences P/N 85-0038 T100 Lot C0038012221853

Validation

These IgGI mouse antibodies were selected for their fluorophores and binding to OneComp eBeads (non-specific binding). Antibodies were used at a final dilution of 1:11 with the OneComp eBeads (5 uL antibody to 50 uL of OneComp eBeads).
https://cytek-web.s3.amazonaws.com/cytekbio.com/Tonbo/TDS/tds-50-0199.pdf
https://cytek-web.s3.amazonaws.com/cytekbio.com/Tonbo/TDS/tds-75-0149.pdf
https://cytek-web.s3.amazonaws.com/cytekbio.com/Tonbo/TDS/tds-85-0038.pdf

Plants

Seed stocks	N/A
Novel plant genotypes	N/A
Authentication	N/A

Flow Cytometry

Plots

Confirm that:

☒ The axis labels state the marker and fluorochrome used (e.g. CD4-FITC).

☒ The axis scales are clearly visible. Include numbers along axes only for bottom left plot of group (a 'group' is an analysis of identical markers).

☒ All plots are contour plots with outliers or pseudocolor plots.

☒ A numerical value for number of cells or percentage (with statistics) is provided.

Methodology

Sample preparation	See online methods
Instrument	rHEALTH ONE, Gallios
Software	rHEALTH Viewer v26.2f_exporthistogram, rHEALTH Capture 59e4, FlowJo v10.8.1
Cell population abundance	N/A
Gating strategy	Gates are outlined in the figures.

☒ Tick this box to confirm that a figure exemplifying the gating strategy is provided in the Supplementary Information.
